# Idiopathic Copper Deficiency Induced Myeloneuropathy

**DOI:** 10.7759/cureus.18130

**Published:** 2021-09-20

**Authors:** Mohammad Mousbah AL-Tabbaa, Emily Horvath

**Affiliations:** 1 Internal Medicine, University of Illinois College Medicine at Peoria, Peoria, USA

**Keywords:** copper, nutritional neurologic symptoms, early copper treatment, copper deficiency, copper myeloneuropathy

## Abstract

Idiopathic nutritional deficiencies are often overlooked in patients with no history of malabsorption. However, it may lead to severe neurologic dysfunction that can sometimes be irreversible. We present a case in which early recognition of copper deficiency has led to a better outcome for the patient, who presented with acute myeloneuropathy.

A 45-year-old male with no significant history of malnutrition or malabsorption presented with complaints of acute encephalopathy, bilateral wrist drop, bilateral tingling and weakness in his hands as well as urinary incontinence. Workup upon arrival was nonrevealing, the patient was treated initially as presumed AIDP (acute inflammatory demyelinating polyradiculopathy), and he underwent plasmapheresis with no response. Since the patient did not respond to plasmapheresis and he had a significantly low folate levels with initial labs. Further nutritional workup was done, which revealed low copper (levels of 0.45), vitamins A, E, and B1. The patient was also tested for celiac which was negative, underwent upper endoscopy and colonoscopy which were both not significant. Decision was made to treat patient early with IV copper infusion as symptoms were deemed most likely due to copper deficiency. The patient received a total of 4 IV doses, after which the patient had a significant clinical response after infusion therapy and repeat copper levels revealed an increase as well (levels of 0.71). Prior to discharge, the patient had significant improvement in wrist drop as well as symptoms of tingling and numbness.

Despite being a trace element, copper deficiency can cause significant neurologic impairment. Furthermore, early recognition has proved to be imperative in neurologic recovery and supplementation has proven to be successful in improving patient’s quality of life.

## Introduction

Nutritional deficiencies are associated with various neurologic syndromes, which have a wide range of clinical presentations depending on the deficient element(s). Most commonly, “deficiencies occur in the context of malnutrition, malabsorption, increased nutrient loss (such as with dialysis), autoimmune conditions such as pernicious anemia, and with certain drugs that inhibit nutrient absorption” [1]. It is infrequently reported that nutritional deficits leading to significant neurologic symptoms are of idiopathic nature. Here we present a striking case report of idiopathic nutritional deficiency related myeloneuropathy (peripheral asymmetric motor and sensory neuropathy). This clinical syndrome was found to be due to deficiencies in copper, vitamin E, folate, vitamin A and thiamine, with copper being the main culprit behind the patient presentation.

## Case presentation

A 45-year-old male with a medical history significant for morbid obesity (body mass index, BMI 53.75) seizure disorder, type 2 diabetes mellitus, chronic kidney disease (CKD) stage IV (reported history of immunoglobulin A, IgA nephropathy), obstructive sleep apnea on home continuous positive pressure machine (OSA on home CPAP) and protein C and antithrombin III deficiencies was transferred to our center for acute encephalopathy, bilateral wrist drop, and polyneuropathy. Upon arrival the patient was found to be somnolent but oriented, and complained of bilateral tingling and weakness in his hands as well as urinary incontinence. Prior to admission the patient was independent at baseline; despite being morbidly obese, the patient was able to perform activities of daily living without assistance. 

Neurologic exam of motor skills upon arrival was significant for decreased bilateral grip strength and decreased bilateral quadriceps strength (right>left). Sensory exam was notable for decreased light touch sensation of upper extremities (right>left), as well as diminished light touch sensation of lower extremities (left>right). Examination of reflexes demonstrated: diminished bilateral biceps, triceps, and brachioradialis response as well as diminished bilateral patellar reflexes and no plantar response (Table 1). A lumbar puncture was performed with no significant findings (Table 2). Plasma exchange therapy was started empirically as a presumptive diagnosis of autoimmune demyelinating polyneuropathy (AIDP) was made. He underwent five sessions of plasma exchange with no clinical improvement. MRI of thoracic spine did not reveal any spinal abnormalities. Electromyography was performed and not supportive of a demyelinating process; however, it was suggestive of mononeuritis multiplex or an acute motor axonal neuropathy.

**Table 1:** Physical exam.

Muscle group	Strength admission	Strength prior to discharge
Right grip	2/5	3/5
Left grip	2/5	3/5
Right elbow flexors	2/5	3/5
Left elbow flexors	2/5	3/5
Right hip flexors	2/5	3/5
Left hip flexors	3/5	4/5
Right plantarflexion	2/5	3/5
Left plantarflexion	2/5	3/5
Right dorsiflexion	2/5	3/5
Left dorsiflexion	2/5	3/5

**Table 2:** CSF analysis. CSF, cerebrospinal fluid

CSF analysis		
Lab	Result	Normal values
Color	Colorless	N/A
Nucleated cells	1/mm3	N/A
Neutrophils	12%	N/A
Lymphocytes	88%	N/A
Protein	25.3 mg/dL	12-60 mg/dL
Glucose	73 mg/dL	40-70 mg/dL
Culture	No growth in two days	N/A

Due to a lack of clinical improvement and a finding of low folate acid values on admission laboratory studies, other vitamin and element levels were measured. The patient was found to have significant deficiencies in copper, vitamin A, vitamin E, and vitamin B1 (Table 3). Further history obtained from the patient revealed no risk factors for malabsorption including proton pump inhibitor use, zinc supplementation, or bariatric surgery. Studies for celiac disease were also negative. There were no abnormal dietary habits, unconventional diets, or excessive alcohol drinking identified. Of note, the patient had undergone a colonoscopy, upper endoscopy, and abdominal CT scan prior to admission, all of which were unremarkable.

**Table 3:** Lab results. IGA, immunoglobulin A; ANA, antinuclear antibody; ANCA, antineutrophil cytoplasmic antibodies; MPO, myeloperoxidase; WBC, white blood cell; HMB, 3-hydroxy-3-methylglutaryl; HGB, hemoglobin; HCT, hematocrit; MCV, mean corpuscular volume; MCH, mean corpuscular hemoglobin; MCHC, mean corpuscular hemoglobin concentration; RDW, red cell distribution width; MPV, mean platelet volume;

Initial labs			Repeat values	
Lab	Result	Normal value	Result	Time interval
Copper	0.45 mcg/mL	0.75-1.45 mcg/mL	0.71 mcg/mL	7 days
Folate	<1.6 ng/mL	7.0-31.4 ng/mL	8.0 ng/mL	9 days
Ferritin	798 ng/mL	22-274 ng/mL	No repeat	
Iron	65 mcg/dL	31-144 mcg/dL	No repeat	
Vitamin A	12.9 mcg/dL	32.5-78.0 mcg/dL	No repeat	
Vitamin B1	36 nmol/L	70-180 nmol/L	No repeat	
Vitamin B12	512 pg/mL	213-816 pg/mL		
Vitamin E	4.8 mg/L	5.5-17.0 mg/L	No repeat	
Vitamin D	35 ng/mL	30-100 ng/mL	No repeat	
Ceruloplasmin	21 mg/dL	20-60 mg/dL	No repeat	
Zinc	0.65 mcg/mL	0.66-1.10 mcg/mL	No repeat	
Paraneoplastic panel	Negative	N/A	N/A	
Celiac panel (IGA, Gliadin IGA antibody, tissue transglutaminase IGA)	Negative	N/A	N/A	
Aldolase	9.0 U/L	<7.7 U/L		
Ganglioside antibodies	Neg	N/A	N/A	
ANA	Negative	N/A	N/A	
ANCA panel (MPO, PR3 and ANCA screen)	Negative	N/A	N/A	
Serum protein electrophoresis	Negative	N/A	N/A	
HMG-CoA Reductase Ab,	<20.0 CU	<20.0 CU	N/A	
WBC	5.45	4.00-12.100 10^3^/mcL	5.45	21 days
HGB	7.6	13.00-16.5 g/dL	8.6	21 days
HCT	24.2	38%-50%	27.4	
MCV	91.3	82-96 fL	94.5	21 days
MCH	28.7	31.0-36.0 g/dL	29.7	21 days
MCHC	31.4	31.0-36.0 g/dL	31.4	21 days
Platelet count	176	140-440 10^3^/mcL	241	21 days
RDW	14.8	11.8-15.5%	16.8	21 days
MPV	9.7	8.0-12.6 fL	9.5	21 days

Nutritional repletion was initiated and the patient was started on copper infusion therapy (receiving a total of four doses and then switched to oral copper), as well as folic acid (which was started one week prior to copper infusion), Vitamin A, Vitamin E, and Vitamin B1 supplementation. Repeat copper levels were increased and essentially within normal range. Most notably, the patient showed clinical improvement with an increase in grip strength as well as decreased tingling and numbness, which was further supported by the decrease in use of PRN (from the latin Pro Re Nata) medications for symptom control as well as a significant improvement in the patient's mental status. 

Neurologic strength exam prior to discharge revealed increased grip strength bilaterally, increased elbow flexion bilaterally, and increased hip flexion bilaterally. Bilateral plantar and dorsiflexion were also improved (Table 1). The patient was discharged to a skilled nursing facility and was instructed to follow up with neurology as well as obtain repeat nutritional labs to determine the length of supplementation. 

Given the fact that the patient did not show any clinical improvement until copper infusion was started, it was deemed to be the main culprit of the patient's presentation. Also of note, the patient had a normal mean corpuscular volume (MCV) anemia (Table 3), which does not correlate with folic acid deficiency. Therefore, the patient's symptoms were deemed most likely secondary to copper deficiency. However, it would be difficult to completely rule out that folic acid may have contributed to the patient's symptoms. 

Upon follow up with the rehab facility two months after discharge, the patient has had continued improvement with significantly increased strength in his bilateral wrists as well as marked reduction in neuropathic pain symptoms. He has demonstrated a significant increase in motor strength and is now able to eat independently.

## Discussion

Nutritional deficiencies are an important source of neurologic dysfunction. Thiamine, folic acid, and other more common vitamin deficiencies are well studied and have a neurologic syndrome associated with each [2]. However, copper deficiency is often initially overlooked and only discovered when conventional therapies of more common neurologic presentations fail to improve clinical symptoms. Recently, copper deficiency has become a more recognized cause of myeloneuropathy. Copper myeloneuropathy was first described in the early 2000s [3], and upon PUBMED literature review, multiple case reports are found describing copper deficiency as a source of myeloneuropathy. Nonetheless, no randomized clinical trial or a large multicenter study was identified.

One study described two cases of idiopathic myeloneuropathy [4] in which the patient history was unremarkable for malabsorption or malnutrition. However, both patients had findings on spinal MRI which were suggestive of myelopathy, and which was not the case in our patient. Both patients improved with oral copper supplementation, with complete resolution of symptoms in one of them. Furthermore, improvements in the MRI abnormalities were demonstrated as well.

No optimal copper dosing recommendations or route of supplementation was identified upon literature search. One study [5] suggests that the choice of route and dosage depends on clinical presentation and severity of the deficiency. In our case, parenteral therapy was initially chosen as copper levels were significantly low and malabsorption was not completely ruled out as a cause of the deficiency. It was also evident in our case that parenteral bridging and rapid correction of serum copper levels may have a role in improving clinical outcome, as our patient had a significant improvement prior to switching to oral supplementation.

Upon literature review it is clear that early identification of copper deficiency is imperative in neurologic recovery. As described in the two cases presented above and, in our case, early recognition played an important role in recovery. On the other hand, one case of optic neuropathy/blindness secondary to copper deficiency was described [6] in which the diagnosis of copper deficiency was delayed, and symptoms did not improve despite copper infusion therapy.

Lastly, a study of 15 copper deficient patients with neurologic manifestation [7] found a statistically significant correlation between copper supplementation and improvement of activities of daily living after 12 months of copper supplementation. These findings support that despite copper being a trace element, supplementation in the setting of clinically significant deficiency improves quality of life.

## Conclusions

Despite being a trace element, copper deficiency can cause significant neurologic impairment. Furthermore, early recognition has proved to be imperative in neurologic recovery and supplementation has proven to be successful in improving the patient’s quality of life. This case emphasizes the wide range of neurologic symptoms that may be present in the setting of copper deficiency, as well as highlights the need to be aware of this important clinical syndrome even when no risk factors may be identified. It is imperative to recognize that this deficiency exists early in a patient’s clinical course to maximize the chance for meaningful neurologic recovery.
